# Redetermination of the crystal structure of di­methyl­bis­[2,4-penta­nedionato(1−)-κ^2^ *O* ^2^,*O* ^4^]tin(IV)

**DOI:** 10.1107/S2056989017003206

**Published:** 2017-03-03

**Authors:** Hans Reuter, Martin Reichelt

**Affiliations:** a Institute of Chemistry of New Materials, University of Osnabrück, Barbarastrasse 7, 49069 Osnabrück, Germany

**Keywords:** crystal structure, redetermination, organotin compound, acetyl­acetonate

## Abstract

The redetermination of the title compound, [Sn(CH_3_)_2_(C_5_H_7_O_2_)_2_] or SnMe_2_(acac)_2_, from CCD data recorded at 100 K basically confirms the previous study based on integrated film data recorded at room temperature [Miller & Schlemper (1972 ▸). *Inorg. Chem.* **12**, 677–681], but reveals a remarkable shrinkage of the *a* axis [7.12 (1) > 6.7694 (4) Å]. The mol­ecule belongs to point group *C_i_* with the Sn^IV^ atom on a centre of inversion. The Sn^IV^ atom shows a slightly distorted octa­hedral coordination sphere with the methyl groups in *trans* positions and a Sn—C bond length of 2.115 (2) Å which may serve as a standard value for an Sn—CH_3_ bond of an octa­hedrally coordinated Sn^IV^ atom. The Sn—O bonds involving the two carbonyl groups of the acetyl­acetonate ligand are of equal length [2.180 (1) and 2.183 (1) Å], as are the C=O [1.273 (1) and 1.274 (1) Å] and C—C bond lengths [1.393 (2) and 1.400 (2) Å]. The acetyl­acetonate ligand deviates considerably from planarity, with a dihedral angle of 5.57 (9)° between the least-squares planes of the two acetone moieties. The four O atoms of the two symmetry-related acetyl­acetonate ligands are arranged in a nearly quadratic rectangle. Weak C—H⋯O inter­actions consolidate the crystal packing.

## Abstract 2

The current redetermination confirms the previous structure report, but with considerably higher precision and accuracy.

## Chemical context   

The crystal structure of the title compound, [Sn(CH_3_)_2_(C_5_H_7_O_2_)_2_] or SnMe_2_(acac)_2_, was determined in the early 1970s at room temperature by visual estimation of film data and refined to a final conventional *R* value of 0.079 (Miller & Schlemper, 1972 ▸). All bond lengths and angles of the original study seem chemically reasonable but accuracy suffers from the limited precision of that kind of data collection. As SnMe_2_(acac)_2_ serves as a reference for all diorganotin(IV) di­acetyl­acetonates and bis-1,3-diketonates in general, we decided to redetermine its structure from CCD data recorded at low temperature. Moreover, the title compound is an excellent candidate for the determination of the Sn—C_Me_ bond length as another reference in case the Sn^IV^ atom is in a well-defined octa­hedral coordination. The precise measurement of this Sn—C distance therefore should supplement the observations of Britton (2006 ▸) who found a significant change in Sn—C bond lengths depending on the organic moiety attached to an Sn^IV^ atom.

## Structural commentary   

The redetermination of the crystal structure of the title compound confirms the former results obtained by Miller & Schlemper (1973 ▸) with respect to the chosen space group (*P*2_1_/*n*) and the constitution of the asymmetric unit comprising half a formula unit with the Sn atom at a crystallographic centre of inversion [Wyckhoff symbol: *b*]. As we performed the X-ray measurement at 100 K, the unit-cell volume is somewhat smaller in comparison with the original room-temperature data which is mainly caused by a considerable change of the *a* axis from 7.12 (1) to 6.7694 (4) Å while changes of all other lattice parameters [*b*_original_ = 13.87 (2), *c*_original_ = 7.69 (1) Å, *β*_original_ = 104.7 (2)°] show a normal temperature-dependent shrinkage.
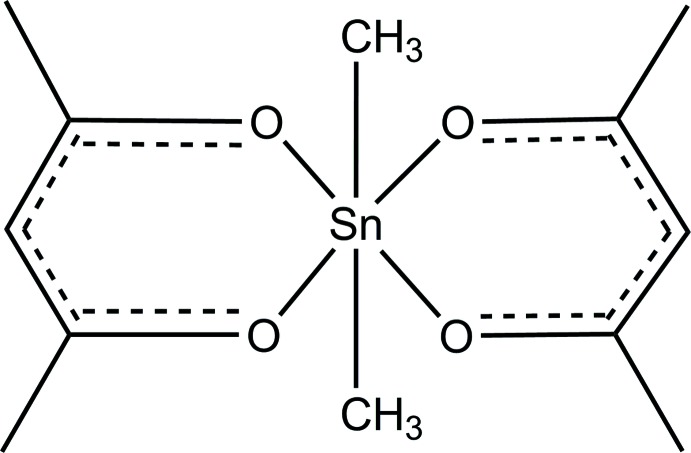


**Figure 1:**
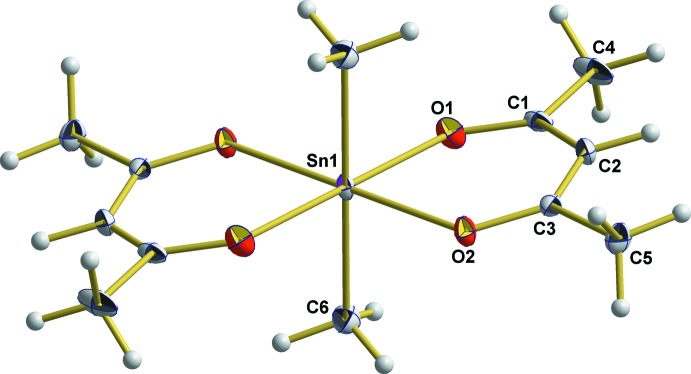
The mol­ecular structure of the title compound, showing the atom-labeling scheme of the asymmetric unit. Displacement ellipsoids are drawn at the 50% probability level.

**Figure 2:**
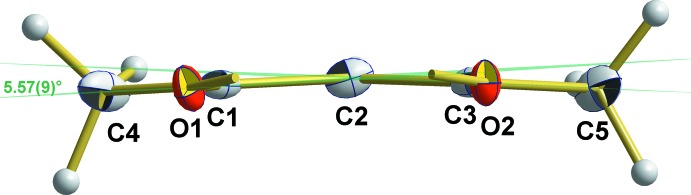
Twisting of the acetyl­acetonate ligand at atom C2 with respect to the least-squares planes (green dashed lines) O1/C1/C2/C5 and O2/C3/C2/C4 in a view parallel to these planes. Non-H atoms are shown as displacement ellipsoids at the 50% probability level.

**Figure 3:**
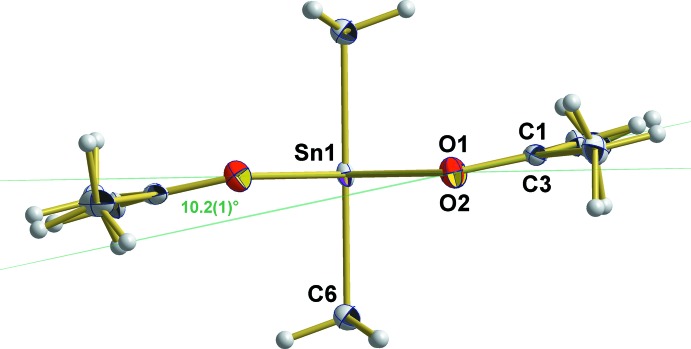
Orientation of the acetyl­acetate ligand (least-squares plane through both carbonyl groups) in relation to the plane defined by the four O atoms coordinating to the Sn^IV^ atom. The view is parallel to these planes (green lines). Non-H atoms are shown as displacement ellipsoids at the 50% probability level.

The Sn atom is octa­hedrally coordinated with the two methyl groups in a *trans* position (Fig. 1 ▸, Table 1 ▸). The Sn—C bond shows a length of 2.115 (2) Å and is significantly shorter than the value [2.14 (2) Å] obtained by Miller & Schlemper (1972 ▸). Because of the higher coordination number, the value differs to some extent from the value of 2.099 (2) Å for Sn with a coordination number of four as observed in di­methyl­dithio­cyanato tin(IV) (Britton, 2006 ▸). The two bonds between the Sn atom and the two different O atoms of the acetyl­acetonate ligand are of equal length [2.180 (1)/2.183 (1) Å]. In accordance with the almost symmetrical bonding of the acetyl­acetonate ligand to tin, C—O [1.273 (2)/1.274 (2) Å] and C—C [1.393 (2)/1.400 (2) Å] bonds of the 1,3-diketonate skeleton are of equal length. Although these values are typical for a delocalized π system, the atoms in question show a significant deviation from planarity at the central C2 atom, resulting in a dihedral angle of 5.57 (9)° between the least-squares planes defined by O1/C1/C2/C3 [deviations from planarity: 0.003 (1), −0.007 (1), 0.002 (1), 0.002 (1) Å, respectively] and O2/C3/C2/C4 [deviations from planarity: 0.002 (1), −0.004 (1), 0.002 (1), 0.001 (2) Å, respectively] (Fig. 2 ▸). Moreover, all bond angles in the six-membered chelate ring are considerably larger [125.61 (9)° at O1, 126.2 (1)° at C1, 128.4 (1)° at C2, 126.07 (13)° at C3, 125.68 (9)° at O2] than expected for *sp* ^2^-hybridized atoms, with exception of the bond angle at Sn1 that amounts to 85.99 (4)°. The four O atoms of the two symmetry-related acetyl­acetonate ligands around the Sn atom form a planar rectangle with similar edge lengths [O1⋯O2 = 2.975 (1)/O1⋯O1 = 3.191 (1) Å], and almost right angles [89.9 (1)° at O1 and 90.1 (1)° at O2]. This plane is nearly perpendicular to the axis through the two methyl groups [deviation: 0.44 (2)°] but constitutes a dihedral angle of 10.2 (1)° with the least-squares plane through the two carbonyl groups of the acetyl­acetonate ligand [deviations from planarity: O1 = 0.006 (1), C1 = −0.007 (1), O2 = −0.006 (1) Å, C3 = 0.007 (1) Å] (Fig. 3 ▸).

## Supra­molecular features   

**Figure 4:**
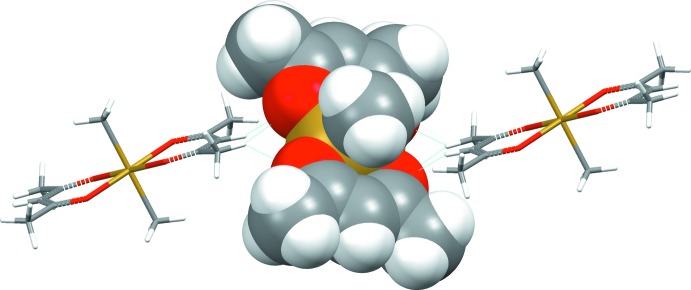
Predominant O⋯H—C contacts (blue dotted lines) of O atoms with the methyl H atoms of the acetyl­acetonate groups of neighbouring mol­ecules. The central mol­ecule is drawn in space-filling mode, while neighbouring mol­ecules are drawn in the stick-model mode visualizing the delocalized π system of the acetyl­acetonate ligands.

**Figure 5:**
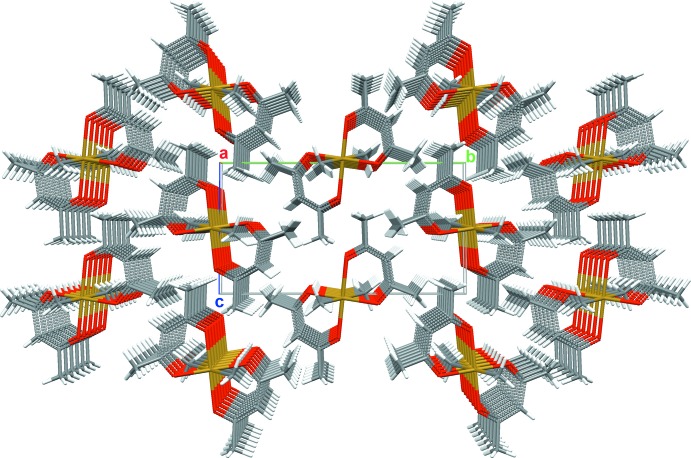
Columnar arrangement of the mol­ecules along the *a* axis.

In the absence of classical H-donor groups, inter­molecular inter­actions are restricted to van der Waals and weak O⋯H—C inter­actions. The most prominent ones are associated with the methyl hydrogen atoms H42 and H51 of the acetyl­acetonate ligand as they inter­act simultaneously with both oxygen atoms of neighbouring mol­ecules [C42⋯O2^i^ = 2.906 Å, C42⋯O1^ii^ = 2.852 Å, C51⋯O2^iii^ = 2.797 Å, H51⋯O1^iv^ = 2.850 Å; symmetry codes: (i) *x*, *y*, 1 + *z*; (ii) 1 − *x*, 1 − *y*,1 − *z*; (iii) 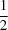 + *x*, 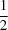 − *y*, 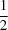 + *z*; (iv) 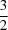 − *x*, −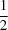 + *y*, 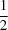 + *z*] (Fig. 4 ▸). These inter­actions are completed by a third O⋯H—C contact of similar length [H2⋯O2^iii^ = 2.893 Å, H43⋯O1^v^ = 2.992 Å, symmetry code: (v) 2 − *x*, 1 − *y*, 1 − *z*] for each oxygen atom. In summary, the inter­molecular contacts result in a columnar arrangement of the mol­ecules parallel to the *a* axis (Fig. 5 ▸).

## Synthesis and crystallization   

The synthesis of the title compound by refluxing a suspension of di­methyl­tin oxide, Me_2_SnO, in acetyl­acetone, acacH, for several hours followed the procedure and experimental details described by McGrady & Tobias (1965 ▸). Single crystals for X-ray diffraction were grown from toluene solution. A suitable single crystal was selected under a polarization microscope and mounted on a 50 μm MicroMesh MiTeGen Micromount^TM^ using FOMBLIN Y perfluoropolyether (LVAC 16/6, Aldrich). The crystals are stable in air.

Spectroscopic data: ^1^H NMR [CDCl_3_, TMS, δ (ppm)], *^n^J* [Hz]): δ(CH_3_—Sn) = 0.58, ^2^ *J*(^1^H—^119/117^Sn) = 100.6/97.1; δ(CH_3_)_acac_ = 1.96; δ(CH)_acac_ = 5.31 (Lockhart & Manders, 1986 ▸; Otera *et al.*, 1981 ▸); ^13^C NMR [CDCl_3_, TMS, δ (ppm)], ^*n*^ *J* [Hz]): δ(CH_3_—Sn) = 7.75, ^1^ *J*(^13^C—^119/117^Sn = 973.7/930.4), δ(CH_3_)_acac_ = 27.94, δ(CH)_acac_ = 100.09, δ(C=O)_acac_ = 190.75 (Lockhart & Manders, 1986 ▸, Otera *et al.*, 1981 ▸); IR [ATR, ν (cm^−1^)]: 3010 *w*, 2920 *w*,1562 *s*, 1512 *s*, 1436 *m*, 1361 *s*,*bd*, 1256 *m*, 1203 *m*, 1015 *m*, 925 *m*, 803 *m*, 781 *m*, 655 *m*, 572 *m*, 552 *m* (McGrady & Tobias, 1965 ▸); Raman [ν (cm^−1^)]: 3092 *w*, 2999 *w*, 2920 *s*, 2708 *w*, 1574 *w*, 1427 *w*, 1366 *m*, 1263 *m*, 1206 *m*, 1194 *m*, 1021 *w*, 927 *m*, 668 *m*, 567 *m*, 512 *s*, 415 *m*, 220 *m*, 130 *m* 94 *m*, 68 *m* (McGrady & Tobias, 1965 ▸).

## Refinement details   

Crystal data, data collection and structure refinement details are summarized in Table 2 ▸. All H atoms were clearly identified in difference Fourier syntheses but were refined assuming idealized geometries and allowed to ride on the carbon atoms with 0.98 Å (–CH_3_), and 0.95 Å (–CH–) and with *U*_iso_(H) = 1.2 and 1.5*U*_eq_(C), respectively.

## Supplementary Material

Crystal structure: contains datablock(s) I, New_Global_Publ_Block. DOI: 10.1107/S2056989017003206/wm5370sup1.cif

Structure factors: contains datablock(s) I. DOI: 10.1107/S2056989017003206/wm5370Isup2.hkl

CCDC reference: 1534819

Additional supporting information:  crystallographic information; 3D view; checkCIF report

## Figures and Tables

**Table 1:** Selected geometric parameters (Å, °)

Sn1—C6	2.115 (2)	O1—C1	1.274 (2)
Sn1—C6^i^	2.115 (2)	O2—C3	1.273 (2)
Sn1—O1	2.180 (1)	C1—C2	1.393 (2)
Sn1—O1^i^	2.180 (1)	C2—C3	1.400 (2)
Sn1—O2	2.183 (1)	C3—C5	1.499 (2)
Sn1—O2^i^	2.183 (1)	C1—C4	1.505 (2)
			
C6^i^—Sn1—C6	180.0	C1—O1—Sn1	125.61 (9)
C6—Sn1—O1	90.20 (5)	O1—C1—C2	126.2 (1)
C6—Sn1—O2	90.41 (5)	C1—C2—C3	128.4 (1)
O1—Sn1—O2	85.99 (4)	C3—O2—Sn1	125.68 (9)

Symmetry code: (i) 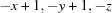.

**Table 2:** Experimental details

Crystal data	
Chemical formula	[Sn(CH_3_)_2_(C_5_H_7_O_2_)_2_]
*M*_r_	346.97
Crystal system, space group	Monoclinic, *P*2_1_/*n*
Temperature (K)	100
*a*, *b*, *c* (Å)	6.7693 (4), 13.8357 (7), 7.6661 (4)
β (°)	104.709 (2)
*V* (Å^3^)	694.46 (7)
*Z*	2
Radiation type	Mo *K*α
μ (mm^−1^)	1.84
Crystal size (mm)	0.29 × 0.23 × 0.16

Data collection	
Diffractometer	Bruker APEXII CCD
Absorption correction	Multi-scan (*SADABS*; Bruker, 2009 ▸)
*T*_min_, *T*_max_	0.616, 0.760
No. of measured, independent and observed [*I* > 2σ(*I*)] reflections	21644, 1672, 1456
*R*_int_	0.024
(sin θ/λ)_max_ (Å^−1^)	0.660

Refinement	
*R*[*F* ^2^ > 2σ(*F* ^2^)], *wR*(*F* ^2^), *S*	0.013, 0.034, 1.11
No. of reflections	1672
No. of parameters	84
Δρ_max_, Δρ_min_ (e Å^−3^)	0.37, −0.27

Computer programs: *APEX2* and *SAINT* (Bruker, 2009 ▸), *SHELXS97* and *SHELXTL* (Sheldrick, 2008 ▸), *SHELXL2014* (Sheldrick, 2015 ▸), *DIAMOND* (Brandenburg, 2006 ▸) and *Mercury* (Macrae *et al.*, 2008 ▸).

## supplementary crystallographic information

### Crystal data

**Table:**

[Sn(CH_3_)_2_(C_5_H_7_O_2_)_2_]	*F*(000) = 348
*M**_r_* = 346.97	*D*_x_ = 1.659 Mg m^−^^3^
Monoclinic, *P*2_1_/*n*	Mo *K*α radiation, λ = 0.71073 Å
*a* = 6.7693 (4) Å	Cell parameters from 9878 reflections
*b* = 13.8357 (7) Å	θ = 2.9–28.5°
*c* = 7.6661 (4) Å	µ = 1.84 mm^−^^1^
β = 104.709 (2)°	*T* = 100 K
*V* = 694.46 (7) Å^3^	Prism, colourless
*Z* = 2	0.29 × 0.23 × 0.16 mm

### Data collection

**Table:**

Bruker APEXII CCD diffractometer	1456 reflections with *I* > 2σ(*I*)
φ and ω scans	*R*_int_ = 0.024
Absorption correction: multi-scan (*SADABS*; Bruker, 2009)	θ_max_ = 28.0°, θ_min_ = 3.0°
*T*_min_ = 0.616, *T*_max_ = 0.760	*h* = −8→8
21644 measured reflections	*k* = −18→18
1672 independent reflections	*l* = −10→10

### Refinement

**Table:**

Refinement on *F*^2^	0 restraints
Least-squares matrix: full	Hydrogen site location: inferred from neighbouring sites
*R*[*F*^2^ > 2σ(*F*^2^)] = 0.013	H-atom parameters not defined?
*wR*(*F*^2^) = 0.034	*w* = 1/[σ^2^(*F*_o_^2^) + (0.0135*P*)^2^ + 0.383*P*] where *P* = (*F*_o_^2^ + 2*F*_c_^2^)/3
*S* = 1.11	(Δ/σ)_max_ = 0.001
1672 reflections	Δρ_max_ = 0.37 e Å^−^^3^
84 parameters	Δρ_min_ = −0.27 e Å^−^^3^

### Special details

**Table:**

Geometry. All esds (except the esd in the dihedral angle between two l.s. planes) are estimated using the full covariance matrix. The cell esds are taken into account individually in the estimation of esds in distances, angles and torsion angles; correlations between esds in cell parameters are only used when they are defined by crystal symmetry. An approximate (isotropic) treatment of cell esds is used for estimating esds involving l.s. planes.

### Fractional atomic coordinates and isotropic or equivalent isotropic displacement parameters (Å^2^)

**Table:**

	*x*	*y*	*z*	*U*_iso_*/*U*_eq_	
Sn1	0.5000	0.5000	0.0000	0.01302 (5)	
O1	0.64915 (18)	0.48470 (8)	0.28616 (14)	0.0219 (2)	
C1	0.7415 (2)	0.40942 (11)	0.36115 (18)	0.0190 (3)	
C2	0.7883 (2)	0.32773 (11)	0.2732 (2)	0.0209 (3)	
H2	0.8493	0.2758	0.3489	0.055 (3)*	
C3	0.7571 (2)	0.31231 (10)	0.0877 (2)	0.0166 (3)	
O2	0.66423 (16)	0.36896 (7)	−0.03729 (13)	0.0183 (2)	
C4	0.8070 (3)	0.41338 (14)	0.5638 (2)	0.0314 (4)	
H41	0.8498	0.3489	0.6114	0.055 (3)*	
H42	0.6924	0.4353	0.6102	0.055 (3)*	
H43	0.9214	0.4585	0.6018	0.055 (3)*	
C5	0.8410 (3)	0.22215 (11)	0.0252 (2)	0.0272 (3)	
H51	0.9046	0.1820	0.1298	0.055 (3)*	
H52	0.9435	0.2394	−0.0397	0.055 (3)*	
H53	0.7300	0.1860	−0.0553	0.055 (3)*	
C6	0.7456 (2)	0.58584 (11)	−0.0348 (2)	0.0236 (3)	
H61	0.8232	0.6103	0.0825	0.053 (4)*	
H62	0.6921	0.6403	−0.1145	0.053 (4)*	
H63	0.8353	0.5466	−0.0887	0.053 (4)*	

### Atomic displacement parameters (Å^2^)

**Table:**

	*U*^11^	*U*^22^	*U*^33^	*U*^12^	*U*^13^	*U*^23^
Sn1	0.01680 (7)	0.01050 (7)	0.01066 (7)	0.00215 (5)	0.00147 (5)	−0.00056 (5)
O1	0.0300 (6)	0.0211 (6)	0.0130 (5)	−0.0013 (4)	0.0026 (4)	−0.0031 (4)
C1	0.0147 (6)	0.0287 (8)	0.0123 (6)	−0.0080 (6)	0.0010 (5)	0.0042 (6)
C2	0.0182 (7)	0.0235 (8)	0.0200 (7)	0.0039 (6)	0.0031 (6)	0.0110 (6)
C3	0.0139 (6)	0.0134 (6)	0.0236 (7)	0.0007 (5)	0.0067 (5)	0.0038 (6)
O2	0.0252 (5)	0.0144 (5)	0.0148 (5)	0.0057 (4)	0.0039 (4)	0.0001 (4)
C4	0.0312 (8)	0.0485 (11)	0.0116 (7)	−0.0168 (8)	0.0001 (6)	0.0044 (7)
C5	0.0273 (8)	0.0173 (7)	0.0403 (10)	0.0081 (6)	0.0143 (7)	0.0045 (7)
C6	0.0228 (7)	0.0191 (7)	0.0284 (8)	−0.0012 (6)	0.0058 (6)	0.0009 (6)

### Geometric parameters (Å, º)

**Table:**

Sn1—C6	2.115 (2)	C3—C5	1.499 (2)
Sn1—C6^i^	2.115 (2)	C1—C4	1.505 (2)
Sn1—O1	2.180 (1)	C4—H41	0.9800
Sn1—O1^i^	2.180 (1)	C4—H42	0.9800
Sn1—O2	2.183 (1)	C4—H43	0.9800
Sn1—O2^i^	2.183 (1)	C5—H51	0.9800
O1—C1	1.274 (2)	C5—H52	0.9800
O2—C3	1.273 (2)	C5—H53	0.9800
C1—C2	1.393 (2)	C6—H61	0.9800
C2—C3	1.400 (2)	C6—H62	0.9800
C2—H2	0.9500	C6—H63	0.9800
			
C6^i^—Sn1—C6	180.0	O2—C3—C2	126.07 (13)
C6^i^—Sn1—O1^i^	90.20 (5)	O2—C3—C5	115.27 (13)
C6—Sn1—O1^i^	89.80 (5)	C2—C3—C5	118.66 (13)
C6^i^—Sn1—O1	89.80 (5)	C3—O2—Sn1	125.68 (9)
C6—Sn1—O1	90.20 (5)	C1—C4—H41	109.5
O1^i^—Sn1—O1	180.0	C1—C4—H42	109.5
C6^i^—Sn1—O2	89.59 (5)	H41—C4—H42	109.5
C6—Sn1—O2	90.41 (5)	C1—C4—H43	109.5
O1^i^—Sn1—O2	94.01 (4)	H41—C4—H43	109.5
O1—Sn1—O2	85.99 (4)	H42—C4—H43	109.5
C6^i^—Sn1—O2^i^	90.41 (5)	C3—C5—H51	109.5
C6—Sn1—O2^i^	89.59 (5)	C3—C5—H52	109.5
O1^i^—Sn1—O2^i^	85.99 (4)	H51—C5—H52	109.5
O1—Sn1—O2^i^	94.01 (4)	C3—C5—H53	109.5
O2—Sn1—O2^i^	180.0	H51—C5—H53	109.5
C1—O1—Sn1	125.61 (9)	H52—C5—H53	109.5
O1—C1—C2	126.2 (1)	Sn1—C6—H61	109.5
O1—C1—C4	114.7 (2)	Sn1—C6—H62	109.5
C2—C1—C4	119.1 (1)	H61—C6—H62	109.5
C1—C2—C3	128.4 (1)	Sn1—C6—H63	109.5
C1—C2—H2	115.8	H61—C6—H63	109.5
C3—C2—H2	115.8	H62—C6—H63	109.5

Symmetry code: (i) −*x*+1, −*y*+1, −*z*.

## References

[bb1] Brandenburg, K. (2006). *DIAMOND*. Crystal Impact GbR, Bonn, Germany.

[bb2] Britton, D. (2006). *Acta Cryst.* C**62**, m93–m94. 10.1107/S0108270106002034 16518039

[bb3] Bruker (2009). *APEX2*, *SADABS* and *SAINT*. Bruker AXS Inc., Madison, Wisconsin, USA.

[bb4] Lockhart, T. P. & Manders, W. F. (1986). *Inorg. Chem.* **25**, 892–895.

[bb5] Macrae, C. F., Bruno, I. J., Chisholm, J. A., Edgington, P. R., McCabe, P., Pidcock, E., Rodriguez-Monge, L., Taylor, R., van de Streek, J. & Wood, P. A. (2008). *J. Appl. Cryst.* **41**, 466–470.

[bb6] McGrady, M. M. & Tobias, R. S. (1965). *J. Am. Chem. Soc.* **87**, 1909–1916.

[bb7] Miller, G. A. & Schlemper, E. O. (1973). *Inorg. Chem.* **12**, 677–681.

[bb8] Otera, J., Hinoishi, T., Kawabe, Y. & Okawara, R. (1981). *Chem. Lett.* **10**, 273–274.

[bb9] Sheldrick, G. M. (2008). *Acta Cryst.* A**64**, 112–122. 10.1107/S0108767307043930 18156677

[bb10] Sheldrick, G. M. (2015). *Acta Cryst.* C**71**, 3–8.

